# Two ghrelin receptor agonists for adults with malnutrition: a systematic review and meta-analysis

**DOI:** 10.1186/s12937-016-0214-5

**Published:** 2016-11-16

**Authors:** Jianhua Su, Jin Geng, Jisheng Bao, Yin Tang, Menglan Liu, Haibin Yu, Yi Han, Wei Huang, Suming Zhou

**Affiliations:** 1 Department of Geriatric Intensive Care Unit, The First Affiliated Hospital, Nanjing Medical University, Guangzhou road 300, Nanjing, 210029 China; 2 Department of Geriatric Medicine, Wuxi NO.2 People’s Hospital, Nanjing Medical University, Zhongshan road 68, Wuxi, 214002 China; 3 Department of Cardiology, Huai’an First People’s Hospital, Nanjing Medical University, Beijingxi road 6, Huai’an, 223300 China

**Keywords:** Ghrelin, Ghrelin receptor agonist, Anamorelin, Malnutrition, Anorexia, Cachexia

## Abstract

**Background:**

Ghrelin receptor agonists have been established to be important in ameliorating the nutritional conditions in patients with malnutrition. However, some studies have reported inconsistent results. We aimed to coalesce the available evidence on the efficacy of ghrelin receptor agonists for the treatment of malnutrition.

**Methods:**

We searched PubMed, the Cochrane Central Register of Controlled Trials, and EMBASE for relevant articles published through March 2016. Studies comparing the efficacy of ghrelin receptor agonists versus placebo in malnourished patients were eligible for inclusion.

**Results:**

A total of 12 studies involving 1377 patients were included. Compared with placebo, ghrelin receptor agonists could increase the energy intake (standard mean difference [SMD] 2.67, 95% confidence interval [CI] 1.48 to 3.85, *P* < 0.001), lean body mass (weighted mean difference [WMD] 0.25 kg, 95% CI 0.07 to 0.42, *P* = 0.006), fat mass (WMD 0.92 kg, 95% CI 0.05 to 1.8, *P* = 0.038), and grip strength (WMD 0.31 kg, 95% CI 0.207 to 0.414, *P* < 0.001) of patients with malnutrition.

**Conclusion:**

Our analysis indicated that ghrelin receptor agonists could improve the poor nutritional state of malnourished patients by increasing their energy intake, ameliorating their irregular body composition and improving their grip strength. However, these results might be less conclusive due to the limited sample sizes and one potential publication that has not been released.

## Introduction

Malnutrition, a condition that is strongly associated with poor prognosis, is a state of nutrition in which a deficiency of energy, protein and micronutrients causes measurable adverse effects on the body composition, function, and clinical outcomes as well as unintentional weight loss [1]. Malnutrition is a common complication of many diseases, such as chronic heart failure [2], chronic obstructive pulmonary disease(COPD) [3], chronic renal failure(CRF) [4], and cancer cachexia [5]. It may also result from other causes of anorexia, sarcopenia or emaciation, including anorexia nervosa [6], functional dyspepsia [7], and ageing [8]. Thus, a single approach can not reverse all aspects of these complicated syndromes and expect to provide prominent benefits. A comprehensive intervention requires the combination of nutritional support, pharmacotherapeutic methods, and exercise [9].

Ghrelin, a compound predominantly secreted by gastric endocrine cells, is an endogenous ligand for the growth hormone secretagogue receptor and has been shown to increase growth hormone(GH) secretion from the pituitary gland [10]. Ghrelin stimulates appetite and food intake and triggers a positive energy balance through GH-dependent mechanisms [11]. However, because the half-life of ghrelin is short and it must be administered by either intravenous or subcutaneous injection [12], the clinical applications of ghrelin are restricted. Several orally active, and selective ghrelin receptor agonists that have a longer half-life than ghrelin were consequently developed, including anamorelin [13], ibutamoren(MK-677) [14], ulimorelin(TZP-101) [15], ipamorelin [16], relamorelin [17], and macimorelin [18]. A number of studies revealed that ghrelin receptor agonists could stimulate appetite and food intake, improve body composition and muscle wasting, and ameliorate the disregulated nutritional condition in malnourished patients. However, some studies have reported inconsistent results [19–23]. In a recent report, Temel et al. demonstrated that anamorelin could significantly increase lean body mass (LBM) but could not significantly enhance the grip strength of patients with cancer cachexia. Additionally, they did not report caloric intake [24].

Therefore, we performed this meta-analysis to confirm the superiority of ghrelin receptor agonist administration compared with placebo in malnourished patients. Our primary outcome was energy intake(EI), and the secondary outcomes were LBM, fat mass(FM), and grip strength(GS).

## Methods

We conducted this meta-analysis in accordance with PRISMA guidelines [25] and the Cochrane Handbook for Systematic Reviews of Interventions [26] following a registered protocol from the PROSPERO database(CRD42016037466).

### Searching strategy

PubMed, the Cochrane Central Register of Controlled Trials (CENTRAL), and EMBASE were electronically searched by independent investigators(JS and JG) to identify any randomized controlled trial (RCT) published through March 2016 that investigated the comparative effects of ghrelin and its analogues versus placebo in patients with malnutrition. We used following search terms embedded in specific files involving the title, keywords, and abstract: “ghrelin”, “ghrelin receptor agonists”, “malnutrition”, “under-nutrition”, “anorexia”, “cachexia”, “weight loss”, and relevant variants of these items. The search strings were constructed with a Boolean operator. We also manually detected any eligible studies among the references of identified papers and several corresponding reviews to include any potential studies, which would guarantee the precision and recall ratio. No language or publication restrictions were imposed. We did not assess the grey literature.

### Identification of studies

The inclusion criteria were described according to the PICOS acronym (participant, intervention, comparison, outcomes of interest, and study design). For population (P), all of the malnourished patients who were treated with ghrelin receptor agonists were included in this study. For intervention(I) and comparison (C), the studies investigated the comparative effects of ghrelin receptor agonists versus placebo. For outcome of interests (O), we accessed the following outcomes: EI, LBM, FM and GS. Regarding the study design (S), only RCT with or without blind methodology were considered.

The exclusion criteria were as follows: healthy volunteers; subjects under 18 years old; patients with either normal nutrition or obesity; a lack of essential information; animal studies; a review, letter or specialist comment; and non-RCT.

### Data extraction

Two independent investigators (JS and YT) extracted the baseline information and essential data of the expected outcomes from each study, including the last name of the first author, publication year, country, sample size of each group, average age and body mass index(BMI) of participants, disease status of the included patients, interventional protocol, follow-up, and reported outcomes of interest. We contacted the authors to acquire any missing data when necessary. Any divergence was resolved by either consensus or consultation with a third author (JB).

### Accessing the quality of methodology

Two independent investigators (HY and ML) were assigned to critically appraise the methodological quality of all eligible studies in accordance with the Cochrane Handbook of Systematic Review of Interventions. Seven indexes were independently assessed, and the following results were crosschecked: randomization sequence generation, allocation concealment, blinding of participants and study personnel, blinding of outcome assessors, incomplete outcome data, selective reporting, and other biases. The risk of each incorporated study was rated as “ high bias risk ” ,“ unclear bias risk ” or “ low bias risk ” according to the extracted information. A third investigator (JB) was assigned to resolve any disagreement.

### Statistical analysis

Stata 12.0 software (Stata Corp, College Station, Texas, USA) was employed to analyse the pooled effect of EI with the SMD and 95% CI, and of the LBM, FM, and GS with WMD and 95% CIs. Heterogeneity was assessed using the χ2-base Q test with a p < 0.10 and the I^2^ test with I^2^ > 50% suggesting significant heterogeneity [27]. We preferentially used the fixed effects model (Mantel-Haenszel method) for pooled analysis [28]; if high heterogeneity was identified, we used the random effects model (DerSimonian and Larid method) [29]. Given the considerable heterogeneity, we also performed sensitivity analyses by excluding each study to evaluate the contribution of the inclusion of studies for heterogeneity. Publication bias was estimated using Egger’s test and funnel plots with the trim and fill method [30, 31], which were also utilized to adjust for publication bias from potential unpublished studies. Statistical significance was considered when a 2-tailed P value was less than 0.05.

## Results

**Fig. 1:**
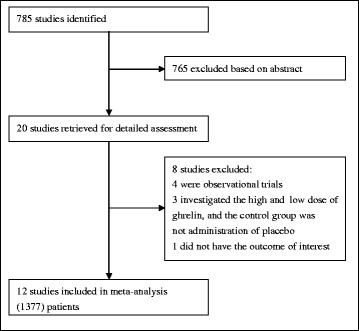
Flow diagram of the retrieval and selection of qualifying literature

Figure 1 shows the flow diagram for study selection. We identified 785 potentially relevant studies based on above search strategy at the initial search stage. After screening the title and abstract, 20 studies were selected for the full-text assessment, and eight trials were excluded due to several reasons such as lack of interested outcomes, ineligible control regimens, and non-RCTs. All selection procedures were performed independently by two investigators. Finally, 12 eligible studies [13, 20, 24, 32–39] were incorporated into this meta-analysis.

### Studies characteristics

The 12 included RCTs comprised 1377 patients, including 1008 male subjects and 369 females. Among the total cohort, 854(62%) patients were assigned to the ghrelin receptor agonists group, and 523(38%) to the control group. The sample size ranged from 14 to 495 subjects, and the follow-up ranged from one day to 12 weeks. All of the included studies compared the efficacy of ghrelin receptor agonists with a corresponding placebo. Nine studies enrolled patients with cancer, while the other three studies did not. Seven studies administered ghrelin, whereas the remaining five trials used anamorelin. The basic characteristics of included studies are shown in Table 1.

**Table 1:** Basic Characteristics of the Included Studies Comparing Ghrelin Receptor Agonists versus Placebo

Study ID	Country	Sample size	Age of participants (Mean ± SD)	Disease	Primary BMI (kg/m^2^, Mean ± SD)	Regimen for the interventional group	Control regimen	Follow up	Reported outcomes
Neary 2004 [36]	UK	7/7	54.3 ± 8.4/54.3 ± 8.4	cancer	22.6 ± 4.1/22.6 ± 4.1	Grelin(5 pmol/kg.min), IVP, single dose	placebo	1 day	EI
Wynne 2005 [38]	UK	9/9	49.8 ± 5.2/49.8 ± 5.2	CRF	24.7 ± 4.4/24.7 ± 4.4	Ghrelin(3.6 nmol/kg), HD, single dose	placebo	3 days	EI
Ashby 2009 [33]	UK	12/12	49.4 ± 14.6/49.4 ± 14.6	CRF	24.3 ± 4.1/24.3 ± 4.1	Ghrelin(12 μg/kg), HD, qd, 7 days	placebo	7 days	EI
Yamamoto 2010 [39]	Japan	10/10	63 ± 6/65 ± 6	cancer	20 ± 2/22 ± 4	Ghrelin(3 μg/kg), IVP, bid, 10 days	placebo	10 days	EI
Shinichi 2010 [32]	Japan	10/10	64.8 ± 10.4/61.6 ± 8.4	cancer	23.1 ± 3.1/24.5 ± 3.8	Ghrelin(3 μg/kg), IVP, bid, 10 days	placebo	10 days	EI, LBM, FM
Miki 2012 [20]	Japan	18/15	70.5 ± 6.2/73.9 ± 6.0	COPD	18.6 ± 2.1/18.0 ± 2.1	Ghrelin(2 μg/kg) , IVP, bid, 3 weeks	placebo	3 week	EI, LBM, FM, GS
Hiura 2012 [35]	Japan	20/20	65.8 ± 5.2/61.8 ± 10.9	cancer	21.6 ± 3/21.0 ± 2.7	Ghrelin(3 μg/kg), IVP, bid, 7 days	placebo	7 days	EI
Garcia 2012 [13]	USA	16/16	61.9 ± 10.29/62.9 ± 8.43	cancer	22.1 ± 3.51/21.6 ± 3.93	Anamorelin, 50 mg, PO, qd, 3 days	placebo	3 days	EI
Garcia 2015 [34]	USA	44/38	65.5 ± NR/65 ± NR	cancer	21.5 ± NR/21.1 ± NR	Anamorelin, 50 mg, PO, qd, 12 weeks	placebo	12 weeks	LBM, FM, GS
Takayama 2016 [37]	Japan	55/60	65.7 ± 8.8/66 ± 9.4	cancer	20.23 ± 3.21/19.80 ± 2.86	Anamorelin, 100 mg, PO, qd, 12 weeks	placebo	12 weeks	LBM, GS
Temel 2016(1) [24]	USA	323/161	63 ± NR/63 ± NR	cancer	23.2 ± 3.6/23.3 ± 3.7	Anamorelin, 100 mg, PO, qd, 12 weeks	placebo	12 weeks	LBM, FM, GS
Temel 2016(2) [24]	USA	330/165	63 ± NR/62 ± NR	cancer	22.5 ± 3.7/22.1 ± 3.7	Anamorelin, 100 mg, PO, qd, 12 weeks	placebo	12 weeks	LBM, FM, GS

*Abbreviations*: *SD* standard deviation, *NR* not reported

### Risk of bias in the included studies

**Fig. 2:**
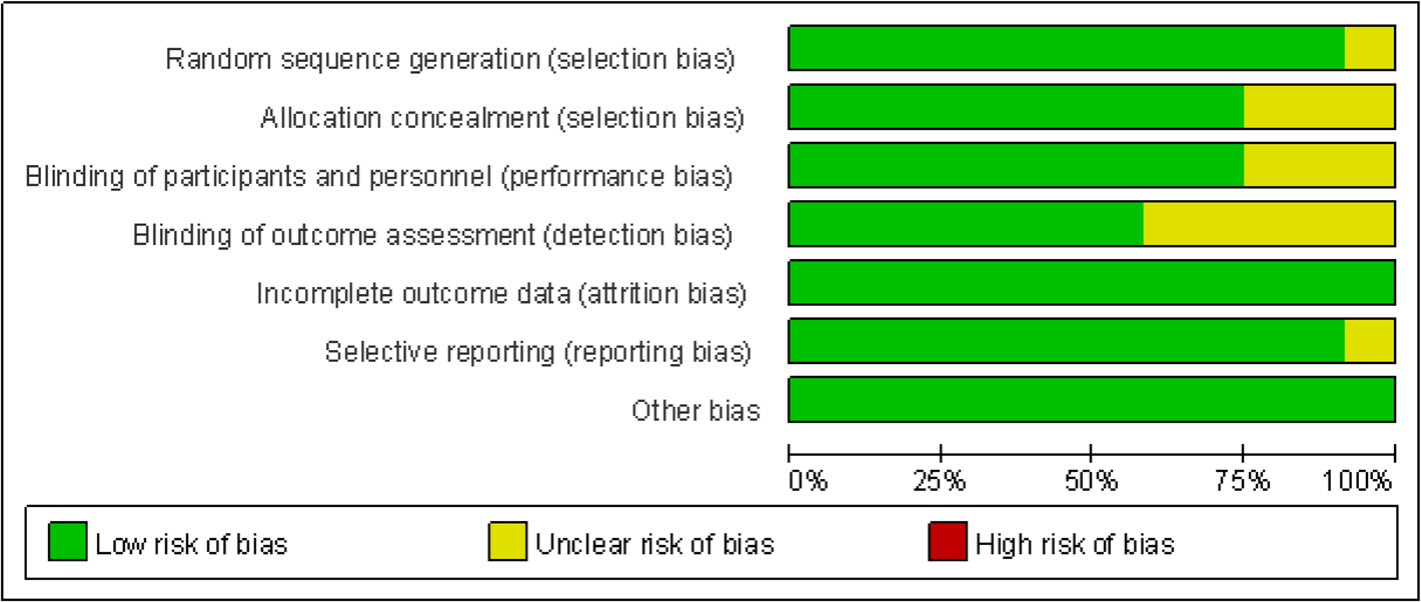
Assessment of the risk of bias: bias of risk graph

**Fig. 3:**
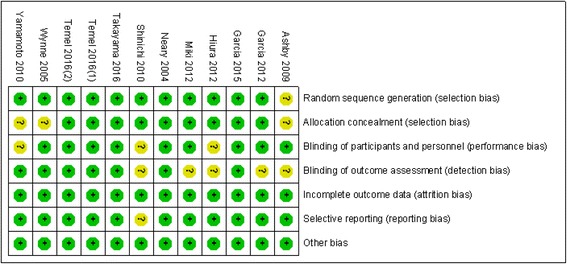
Assessment of the risk of bias: bias of risk summary

We carefully appraised the methodological quality of the included studies according to the Cochrane Collaboration’s Risk of Bias Tool. Four studies provided detailed information regarding the seven total indexes. In the remaining studies, varying degrees of methodological bias were identified. All of the included trials were rated as low bias risk regarding incomplete outcome data because the authors described the drop-out reasons in detail and used the intent-to-treat method to analyse the data. No other bias sources were identified. The graphical results of the methodological quality are shown in Figs. 2 and 3.

### Energy intake

**Fig. 4:**
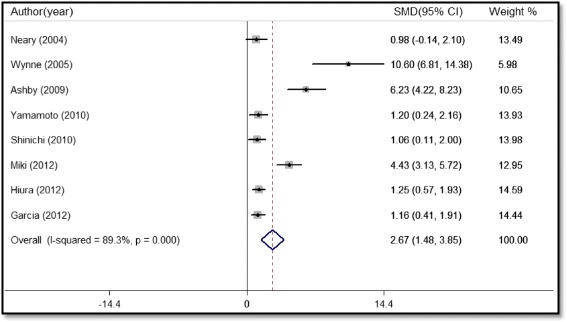
Forest plot of energy intake

Eight studies reported the EI data: seven measured the total calories of ingested food, whereas the eighth estimated the total calories by using a calorie count. In total, 201 participants were included for the pooled analysis, which showed a significantly increased EI after administration of ghrelin receptor agonists (SMD 2.67, 95%CI 1.48 to 3.85, P < 0.001, Fig. 4). There was significant heterogeneity among the studies (I^2^ = 89.3%, P < 0.001). Subgroup analyses showed that the increased EI after the administration of ghrelin receptor agonists remained evident irrespective of the different areas, diseases, therapeutic drug, and follow-up.

Sensitivity analyses showed no alterations of the main outcome after eliminating each study. However, we found no significant heterogeneity when we excluded the studies that did not include cancer patients(I^2^ = 0%, P = 0.99) with a new pooled SMD of 1.12 (95%CI 0.74 to 1.50, P < 0.001).

**Fig. 5:**
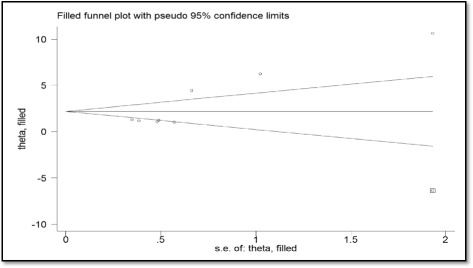
Funnel plot with the trim and fill method for energy intake

Egger’s test was statistically significant (P = 0.037) and visual inspection of the funnel plot seemed to be asymmetric. The trim and fill method suggested that there might be one unpublished studies (Fig. 5). Using trim and fill method, we found that our finding remained significant after adjusting for one unpublished study(SMD 2.18, 95%CI 0.913 to 3.448, P = 0.001).

### Lean body mass

**Fig. 6:**
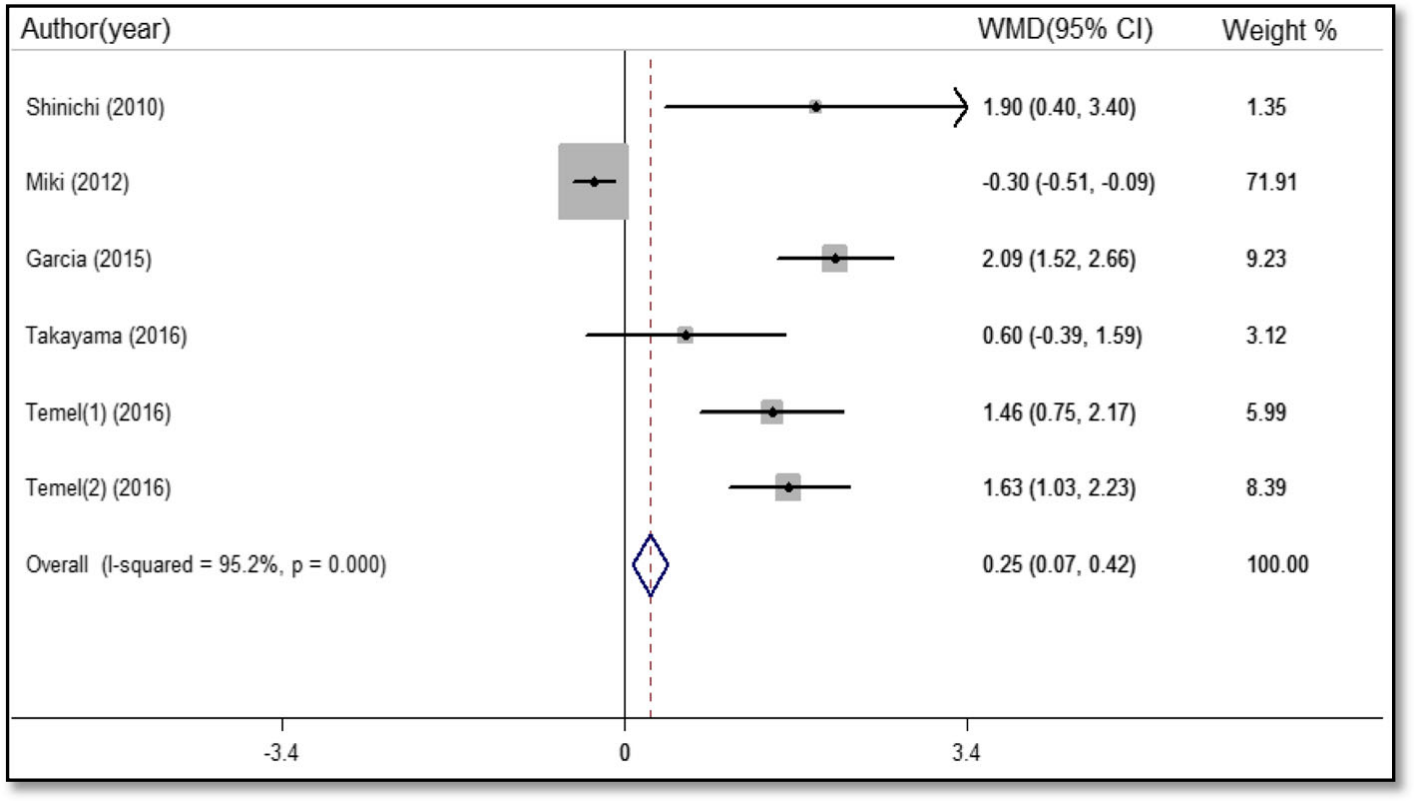
Forest plot of lean body mass

A total of six studies comprising 1178 participants reported LBM data as measured by dual energy x-ray absorptiometry(DEXA). The meta-analysis showed that administration of ghrelin receptor agonists could increase the LBM in malnourished patients (WMD 0.25 kg, 95%CI 0.07 to 0.42, P = 0.006, Fig. 6). There was also significant heterogeneity among the studies regarding LBM (I^2^ = 95.2%, P = 0.000). The subgroup analyses showed that increased LBM after administration of ghrelin receptor agonists remained evident irrespective of the different regions, diseases, or therapeutic drug. Sensitivity analyses showed no alterations of the main outcome after eliminating each study. However, we found no significant heterogeneity when excluded the study by Miki (I^2^ = 43%, P = 0.14) with a pooled WMD of 1.64 kg (95%CI 1.31 to 1.97, P < 0.001). Egger’s test revealed no statistical significance (P = 0.054), indicating that there is no publication bias.

### Fat mass

**Fig. 7:**
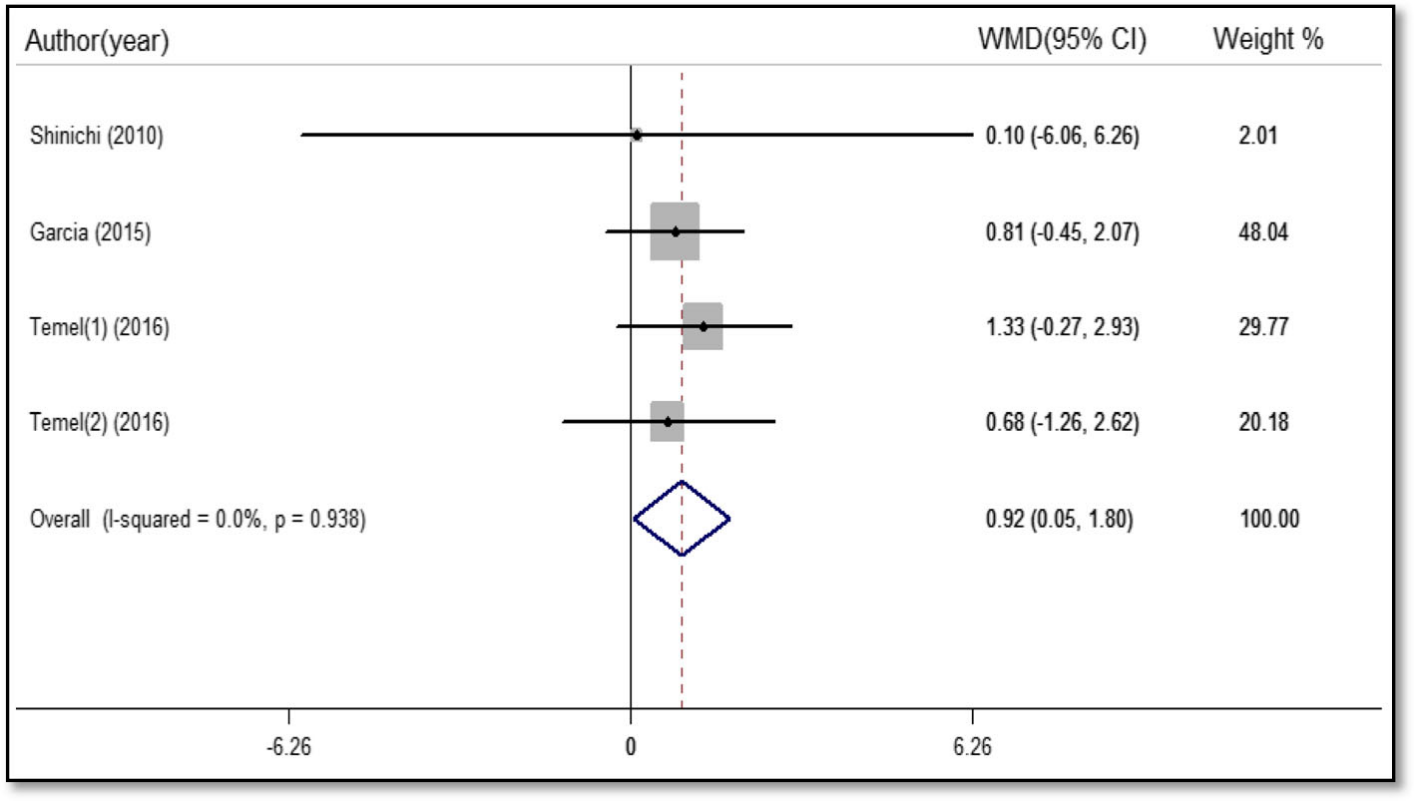
Forest plot of fat mass

There were four studies(923 subjects) that reported FM data, which was also measured by DEXA. The meta-analysis revealed that administration of either ghrelin or anamorelin to malnourished patients could increase the FM (WMD 0.92 kg, 95%CI 0.05 to 1.8, P = 0.038) with no evidence for heterogeneity across the studies (I^2^ = 0%, P = 0.938, Fig. 7). We found no change of the pooled estimate effect and heterogeneity after sensitivity analyses. Egger’s test revealed no statistical significance (P = 0.633), showing that there is no publication bias.

### Grip strength

**Fig. 8:**
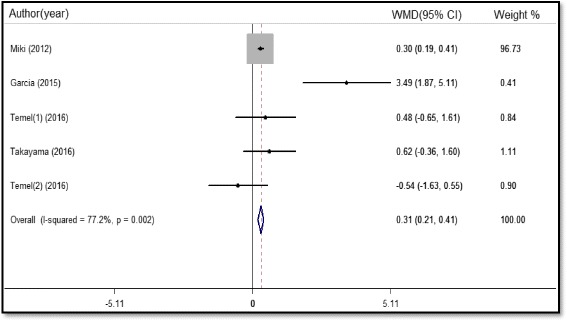
Forest plot of grip strength

A total of five studies comprising 1085 participants reported GS data: two of the studies measured handgrip strength on each side by using a hand-held dynamometer, while the other three only measured the handgrip strength of the non-dominant hand. The meta-analysis showed that administration of ghrelin receptor agonists could increase the GS in malnourished patients (WMD 0.31 kg, 95%CI 0.21 to 0.41, P < 0.001, Fig. 8). Sensitivity analyses showed no alterations of the main outcome after eliminating each study. However, we found no significant heterogeneity when we excluded the study by Garcia (I^2^ = 0%, P = 0.43), with a pooled WMD of 0.3 kg (95%CI 0.19 to 0.4, P < 0.001). Egger’s test revealed no statistical significance (P = 0.501), indicating that there is no publication bias.

## Discussion

In the present study, we demonstrated that administration of ghrelin receptor agonists could ameliorate the nutritional condition of patients with malnutrition by increasing their food intake, changing their body composition, and enhancing their muscle strength. These conclusions were strengthened by the fact that sensitivity analyses and the trim and fill method did not alter these outcomes, although there might be lack of power to detect differences with the limited sample size and other potential publications. There was significant heterogeneity in the pooled analyses of EI and LBM. Therefore, we used a subgroup analysis and sensitivity analysis to identify the causes of heterogeneity. When we excluded the studies that had no cancer patients enrolled, the high degree of heterogeneity vanished. Thus, the differences in patients’ diseases might be the cause of the significant heterogeneity.

Loss of appetite appears in many patients with severe malnutrition, which is not only frequent, but is also associated with a poor prognosis and reduced quality of life [40]. In this study, we found that administration of ghrelin receptor agonists could increase energy intake, which confirms their well-established role in stimulating appetite and increasing food intake [41, 42]. Administration of exogenous ghrelin administration was first noted to promote food intake and weight gain in rats [43, 44]. These effects were independent of the effects of ghrelin on GH stimulation and the GH secretagogue receptor(GHS-R) and might be related to neuropeptide Y(NPY), as indicated by the increase of plasma NPY levels after IV bolus of ghrelin [45]. Another possible explanation is that ghrelin could inhibit the production of the anorectic proinflammatory cytokines such as interleukin-1β, interleukin-6, and tumour necrosis factors, as well as induce secretion of the anti-inflammatory cytokine interleukin-10 [46]. In contrast, in a randomized, double-cross-over clinical study, Strasser et al. used different doses of ghrelin in cancer patients and found that the nutritional intake did not increase following ghrelin administration [19]. Moreover, a single ghrelin infusion could not increase the food intake in patients with postvagotomy diarrhoea [22] and anorexia nervosa [23], although repeated infusions could stimulate hunger and food intake in these subjects [6, 32]. Thus, we postulated that the dose and frequency of ghrelin administration to the enrolled subjects would influence the actual clinical effect. Interestingly, the increase in energy intake following ghrelin administration occurred without compensatory undereating [47] or evidence of tachyphylaxis [33]. However, the included studies evaluated EI by different methods, so we used SMD as the effect magnitude, which was less persuasive than WMD.

Changes in body composition that develop with chronic diseases are usually considered unwanted and are associated with the loss of muscle, fat mass, or both [48]. The loss of lean and fat tissue may be associated with weight loss; such involuntary weight loss has been termed cachexia [49]. Activation of the GHS-R exerted anabolic functions and could lead to weight gain [41]. Ghrelin receptor agonists could increase bodyweight by not only stimulating appetite but also by decreasing energy expenditure, the latter of which was relevant to cancer cachexia [50]. Our results validated the capacity of ghrelin receptor agonists in improving the body composition of patients with under-nutrition as indicated by the significant increase in LBM and FM. Low LBM was associated with a poor prognosis and could predict the toxic effects of treatment [51]. Increased FM reflected improved energy balance, although GH secretion induced by ghrelin receptor agonists may contribute to low adiposity [52]. Thus, the present study illuminated the beneficial effect of ghrelin receptor agonists on the positive changes in body composition and weight gain, which would be promising for the treatment of malnutrition, as losses in body weight and sarcopenia are characteristic features of under-nutrition [53].

Theoretically, a valid treatment for cachexia-associated muscle wasting should increase muscle mass and, as a consequence, enhance muscle strength. However, muscle wasting in cachectic patients results from a variety of factors such as age-related muscle atrophy, therapies targeting the primary disease, comorbidity, and persistent systematic inflammation [24]. Contrary with corticosteroids [54] and progestational drugs [55], which were commonly prescribed as orexigenic agents and could only increase appetite and body weight, our findings verified that ghrelin receptor agonists could significantly enhance muscle strength. Activation using ghrelin receptor agonists induced GH secretion, thereby increasing the insulin-like growth factor-1 (IGF-1) concentration; GH and IGF-1 could promote muscle growth through a direct effect on the muscle and indirectly activating the production of both muscle-restricted IGF-1 and anti-cachectic cytokines [56]. However, our included studies investigated muscle function by measuring handgrip strength with either the dominant or non dominant hand, which measured upper-extremity strength and might not indicate a comprehensively physiological performance. Further studies using internationally validated methods to assess muscle strength are warranted.

An important concern with regard to the application of ghrelin receptor agonists in cancer cachexia is that they may increase the levels of growth factors such as GH and IGF-1 to promote tumour growth. In addition, ghrelin itself may have mitogenic potential. Northrup et al. evaluated ghrelin and anamorelin on tumour growth in mouse models of lung cancer, and found that neither compound could effect tumour growth until the end of the intervention despite the significantly increased GH and IGF-1 levels [57]. Additionally, clinical studies with anamorelin [58] and ghrelin [59] have also shown no significant effect on overall survival compared with placebo. Long-term, large-scale clinical trials are required to determine whether treatment with ghrelin receptor agonists could stimulate tumour growth [60].

There are some limitations in this meta-analysis. First, the basic characteristics of the included patients differed in some confounders. However, sensitivity analysis and the trim and fill method did not alter the results of our primary outcome, which lessened the adverse effect due to this limitation. Second, the sample size of included studies was small, the follow-up was short, and only two ghrelin receptor agonists were analysed among the variety of agonists in existence. Third, there were more male patients than females in each group, so the conclusions were less conclusive for female subjects. Further studies with more subjects, a longer follow-up period and implementation of different ghrelin receptor agonists are required to identify whether the administration of ghrelin receptor agonists would lead to long-term benefits such as reduced total medical cost, decreased hospital stay, and elevated quality of life and overall survival.

## Conclusion

In conclusion, based on the results of our meta-analyses, we confirmed that administration of ghrelin receptor agonists could have beneficial effects on patients with malnutrition. This novel approach, which directly targets appetite stimulation, could have an important role in the future management and prevention of under-nutrition for malnourished patients.

## Abbreviations

BMI: Body mass index
CI: Confidence interval
COPD: Chronic obstructive pulmonary disease
CRF: Chronic renal failure
DEXA: Dual energy x-ray absorptiometry
EI: Energy intake
FM: Fat mass
GH: Growth hormone
GHS-R: GH secretagogue receptor
GS: Grip strength
IGF-1: Insulin-like growth factor-1
LBM: Lean body mass
NPY: Neuropeptide Y
NR: Not reported.
RCT: Randomised controlled trial
SD: Standard deviation
SMD: Standard mean difference
WMD: Weighted mean difference
